# Protective Capacity of the Human Anamnestic Antibody Response during Acute Dengue Virus Infection

**DOI:** 10.1128/JVI.01096-16

**Published:** 2016-11-28

**Authors:** Meihui Xu, Roland Züst, Ying Xiu Toh, Jennifer M. Pfaff, Kristen M. Kahle, Edgar Davidson, Benjamin J. Doranz, Sumathy Velumani, Farhana Tukijan, Cheng-I Wang, Katja Fink

**Affiliations:** a Singapore Immunology Network, Agency for Science Technology and Research, Singapore; b Integral Molecular, Philadelphia, Pennsylvania, USA; c School of Biological Sciences, Nanyang Technological University, Singapore; University of Southern California

## Abstract

Half of the world's population is exposed to the risk of dengue virus infection. Although a vaccine for dengue virus is now available in a few countries, its reported overall efficacy of about 60% is not ideal. Protective immune correlates following natural dengue virus infection remain undefined, which makes it difficult to predict the efficacy of new vaccines. In this study, we address the protective capacity of dengue virus-specific antibodies that are produced by plasmablasts a few days after natural secondary infection. Among a panel of 18 dengue virus-reactive human monoclonal antibodies, four groups of antibodies were identified based on their binding properties. While antibodies targeting the fusion loop of the glycoprotein of dengue virus dominated the antibody response, two smaller groups of antibodies bound to previously undescribed epitopes in domain II of the E protein. The latter, largely serotype-cross-reactive antibodies, demonstrated increased stability of binding at pH 5. These antibodies possessed weak to moderate neutralization capacity *in vitro* but were the most efficacious in promoting the survival of infected mice. Our data suggest that the cross-reactive anamnestic antibody response has a protective capacity despite moderate neutralization *in vitro* and a moderate decrease of viremia *in vivo*.

**IMPORTANCE** Antibodies can protect from symptomatic dengue virus infection. However, it is not easy to assess which classes of antibodies provide protection because *in vitro* assays are not always predictive of *in vivo* protection. During a repeat infection, dengue virus-specific immune memory cells are reactivated and large amounts of antibodies are produced. By studying antibodies cloned from patients with heterologous secondary infection, we tested the protective value of the serotype-cross-reactive “recall” or “anamnestic” response. We found that results from *in vitro* neutralization assays did not always correlate with the ability of the antibodies to reduce viremia in a mouse model. In addition, a decrease of viremia in mice did not necessarily improve survival. The most protective antibodies were stable at pH 5, suggesting that antibody binding in the endosomes, after the antibody-virus complex is internalized, might be important to block virus spread in the organism.

## INTRODUCTION

Multiple studies have characterized the human antibody (Ab) response to natural dengue virus (DENV) infection based on monoclonal antibodies (MAbs) that were isolated from plasmablasts during the acute phase of infection or from memory B cells after recovery (1–7). However, antibody-associated correlates of protection and mechanisms of neutralization that prevent or reduce the spread of the virus in the organism are still poorly understood. This was best illustrated by the recent clinical trials of the leading vaccine from Sanofi-Pasteur, for which the overall efficacy across all four DENV serotypes was only 60.3% despite generally high neutralizing titers in vaccinees (8). Vaccine efficacy by serotype placed DENV serotype 2 (DENV-2) at the bottom, with a reported efficacy of only 43% (8). Interestingly, vaccine efficacy was higher in children above the age of 9 years, and efficacy was associated with seropositivity, suggesting that the protective mechanisms of the vaccine are related to the reactivation of specific immune memory cells, or the so-called anamnestic response.

The aim of this study was to address the protective capacity of antibodies produced during a natural anamnestic response after symptomatic reinfection with a heterologous serotype of DENV.

The current literature focuses largely on the description of epitopes of potently neutralizing antibodies. In turn, immunodominant epitopes that elicit weakly neutralizing or nonneutralizing antibodies and their possible functions and implications for overall disease resolution, or enhancement, have rarely been described. The envelope (E) glycoprotein is the surface protein of DENV particles and is the primary target of the humoral immune response, eliciting neutralizing antibodies that are necessary to prevent reinfection (9). Antibodies against the E glycoprotein have been shown to inhibit virus attachment and infection *in vitro*, and passive transfer of E glycoprotein-specific antibodies protected mice from dengue virus challenge (10, 11).

The tertiary structure of the E glycoprotein has three domains, EDI, -II, and -III, which fold from a discontinuous primary protein sequence. EDI forms a central β-barrel linking EDII to EDIII (12). EDII contains a dimerization region that is responsible for the spontaneous dimer formation of E proteins. EDII also contains a fusion loop that is necessary for membrane fusion with host cells during the infection process. EDIII assumes an immunoglobulin-like fold and mediates host cell receptor binding, and consequently, antibodies against EDIII have been shown to possess potent type-specific neutralization capacities (13). However, dengue virus has the capacity to escape from these antibodies by mutating EDIII (10, 14, 15). A total of 90 E homodimers assume a “herringbone” configuration on the mature virus surface. The E glycoprotein undergoes several drastic conformational changes during the infection cycle to enable host cell infection and production of new virus progeny (16).

Dengue virus enters susceptible host cells through receptor-mediated endocytosis. Acidification in the endosome promotes dissociation of homodimers into monomers, followed by an irreversible reassociation of the monomers to form the fusogenic trimer structure that exposes the fusion peptide. The fusogenic trimer structure extends outward from the virion surface toward the host cell membrane to facilitate membrane fusion, releasing the viral genome into the host cell's cytoplasm and triggering translation of nonstructural and structural viral proteins and propagation of the viral genome (16). Consequently, epitope accessibility on the E protein is dependent on the pH of the cellular compartment in which the virus is potentially bound by an antibody. The pH stability of an antibody can therefore be an important factor determining its protective capacity. Here, we provide new insight into the correlation of the epitope, *in vitro* neutralization, pH-dependent antibody stability, and *in vivo* protective capacity of human plasmablast-derived antibodies.

## MATERIALS AND METHODS

### Monoclonal antibodies and virus strains.

The panel of human monoclonal antibodies used in this study has been described previously (7). The antibody sequences are available in GenBank (see Table S1 in the supplemental material). The humanized mouse monoclonal antibody 4G2 was a kind gift from Brendon John Hanson, DSO Laboratories, Singapore. Antibodies 747(4)A11 and 752-2 C8 were produced according to the published sequences. All dengue virus strains used in this study were propagated in C6/36 mosquito cells. The DENV stocks used were Western Pacific 74 [ U88535.1] or DENV-1-D1/SG/05K2916DK1/2005 [EU081234.1], TSV01 [AY037116.1] or DENV-2/SG/D2Y98P-PP1/2009, (JF327392.1), VN32/96 [EU482459], and 2641Y08 [HQ875339.1] for dengue virus serotypes 1, 2, 3, and 4, respectively.

### ELISA and competition binding assays.

Whole virus particle enzyme-linked immunosorbent assay (ELISA) was performed by capturing virions from infected C6/36 cell supernatant on 4G2-coated plates. For binding to recombinant E (rE), MaxiSorp plates were coated with 150 ng of purified rE protein in 100 μl coating buffer at 4°C overnight. rE protein was produced in S2 cells as described previously (17). Antibodies were added at 1 μg/ml, and binding was detected by adding anti-human IgG-horseradish peroxidase (HRP). For competition ELISAs, biotinylated antibodies were added to the plates at 0.01 μg/ml, while the competing nonlabeled antibodies were added in 100-fold excess. Binding was detected by adding streptavidin-HRP and developed by adding 3,3′,5,5′-tetramethylbenzidine (TMB) substrate. The reaction was stopped by adding 1 M HCl. The optical density at 450-nm wavelength (OD_450_) was measured on an Enspire plate reader.

### Neutralization assays (plaque reduction neutralization test [PRNT]/fluorescence-activated cell sorter [FACS]).

BHK-21 cells were seeded in each well of a 24-well plate and incubated at 37°C overnight. Antibodies were diluted 4-fold over six dilution steps, starting from 30 μg/ml, and the antibody dilutions were then added to a constant amount of virus (multiplicities of infection [MOI] of 0.01 for DENV-1 strain 05K2916 [DENV-1-05K2916] and 0.1 for DENV-2-TSV01, DENV-3-VN32, and DENV-4-2641Y08). The virus-antibody mixtures were incubated at 37°C for 1 h prior to infection of cells. A methylcellulose overlay was added, and the plates were incubated for 5 days before plaque visualization by crystal violet staining. The flow cytometry-based neutralization assay was performed with U937 cells stably expressing DC-SIGN. The number of infected cells was determined by staining all the cells intracellularly with 4G2-AlexaFluor 647 and anti-NS1-Alexa 488 as described previously (7). The MOI used were 0.1 for DENV-1-05K2916, 0.5 for DENV-2-TSV01, 0.2 for DENV-3-VN32, and 5 for DENV-4-2641Y08. The 50% plaque reduction neutralization titer (PRNT_50_) and 50% effective concentration (EC_50_) values, respectively, were defined as the concentration of antibody that results in a 50% reduction of plaques or infected cells, and these values were calculated using a three-parameter nonlinear curve fitted in GraphPad Prism software.

### Epitope-mapping studies on recombinant protein.

DENV-2 E protein mutants were produced with a QuikChange site-directed mutagenesis kit (Agilent). A V5 tag at the C terminus of the E protein was used to facilitate immobilization of E protein dimers on ELISA plates (see Fig. S1 in the supplemental material for validation). E proteins were produced in S2 cells and purified as described previously (18). ELISA plates were coated with polyclonal rabbit anti-V5 antibody, and individual E protein mutants were added at a concentration of 5 μg/ml in 50 μl coating buffer (half-area ELISA plates; Greiner). After blocking with 3% skim milk and 0.05% Tween 20 in phosphate-buffered saline (PBS), individual antibodies were added at a concentration of 1 μg/ml. Bound antibodies were detected with anti-human IgG-HRP antibody (Sigma), and TMB was used as a substrate for color development.

Antibody epitopes in the postfusion trimeric E protein were illustrated on the DENV-2 structure (Protein Data Bank [PDB] ID 3G7T) using Yasara software.

### Shotgun mutagenesis epitope mapping.

Shotgun mutagenesis epitope mapping (19) was performed using comprehensive mutation libraries obtained by subjecting DENV-3 (strain CH53489) and DENV-4 (strain 341750) prM/E expression constructs to high-throughput mutagenesis. Random mutations were introduced into the DENV-3 prM/E polyprotein, while for DENV-4, each prM/E residue was mutated to alanine (and alanines to serine). The mutant plasmids were arrayed in 384-well plates (one mutation per well), transfected into HEK-293T cells, and allowed to express for 22 h. The cells were monodispersed using Cell Stripper (Cellgro), fixed in 4% (vol/vol) paraformaldehyde (PFA), permeabilized for 20 min with 0.1% (wt/vol) saponin (Sigma-Aldrich) in PBS plus calcium and magnesium (PBS++), and then stained for 1 h with purified antibodies diluted in 10% normal goat serum (NGS) (Sigma)-0.1% saponin, pH 9. The primary antibody concentrations were selected using an independent immunofluorescence titration curve against wild-type prM/E to ensure that the signal was within the linear range of detection. Antibodies were detected by incubating with AlexaFluor 488-conjugated secondary antibody (3.75 μg/ml; Jackson ImmunoResearch Laboratories) in 10% NGS-0.1% saponin for 30 min. The cells were washed 3 times with PBS++-0.1 saponin, followed by 2 washes in PBS, and the mean cellular fluorescence was detected using a high-throughput flow cytometer (HTFC) (Intellicyt). Antibody reactivities against each mutant clone were calculated relative to wild-type prM/E protein reactivity by subtracting the signal from mock-transfected controls and normalizing it to the signal from wild-type prM/E-transfected controls. Mutations within critical clones were identified as critical to the antibody epitope if they did not support reactivity of the test antibody but did support reactivity of other control antibodies. This counterscreen strategy facilitates the exclusion of prM/E mutants that are locally misfolded or have an expression defect. Control antibodies were selected to represent epitopes over diverse regions on the E protein. The use of control MAbs to confirm the folding of each mutant, combined with mapping each mutant directly in human cells that correctly process prM/E, provides confidence that identified epitope residues are affecting the binding epitope directly and not having indirect effects. Critical amino acids required for antibody binding were visualized on a DENV-2 or DENV-3 Env crystal structure (PDB ID 1OAN and 1UZG).

### pH stability assay.

Goat anti-human IgG was immobilized on a GLC sensor chip (Bio-Rad) by amine coupling. Antibody analytes were allowed to bind to the immobilized anti-human IgG before the introduction of rE protein. Measurements were recorded at 25°C in running buffer at pH 5 or pH 7 on ProteOn XPR36 equipment (Bio-Rad).

The sandwich ELISA described in “Epitope-mapping studies on recombinant protein” above was modified as follows. E proteins were buffer exchanged with MES (morpholineethanesulfonic acid) buffer, pH 5, before adding them to the anti-V5-tag-coated plates. After blocking, the antibodies were diluted in MES buffer, pH 5, and incubated for 2 h at room temperature. For pH 7 measurements, E protein in PBS, pH 7.4, was used instead of MES buffer.

### Immunofluorescent staining.

Antibodies (1 μg/ml) were incubated with DENV-1 (or DENV-2 [data not shown]) for 1 h at 37°C. BHK-21 cells were grown on chamber slides (ibidi). Antibody-virus mixtures were added to the BHK-21 cells on ice for 20 min for synchronization of antibody-virus uptake by the cells. The chambers were moved to 37°C for 7 min. The cells were then fixed with 2% PFA, permeabilized, and stained with rabbit anti-EEA Ab. Anti-human IgG AF488 and goat anti-rabbit IgG AF568 (both from Molecular Probes) were used to detect the primary antibodies. Hoechst was used to stain nuclei. Photographs were taken at ×100 magnification with an Olympus confocal microscope.

### Protection assay in mice.

AG129 mice were treated with 100 μg purified antibody injected intravenously (i.v.) 24 h prior to virus challenge, which was administered intraperitoneally. Blood was collected 3 days postinfection, and viremia was quantified by TaqMan reverse transcription (RT)-PCR (20). The animal experiments were conducted according to the rules and guidelines of the Agri-Food and Veterinary Authority and the National Advisory Committee for Laboratory Animal Research, Singapore. The experiments were reviewed and approved by the Institutional Review Board of the Biological Resource Center, Singapore (Institutional Animal Care and Use Committee; protocol 151099).

## RESULTS

### Binding properties identify four main antibody groups in the anamnestic response.

**FIG 1:**
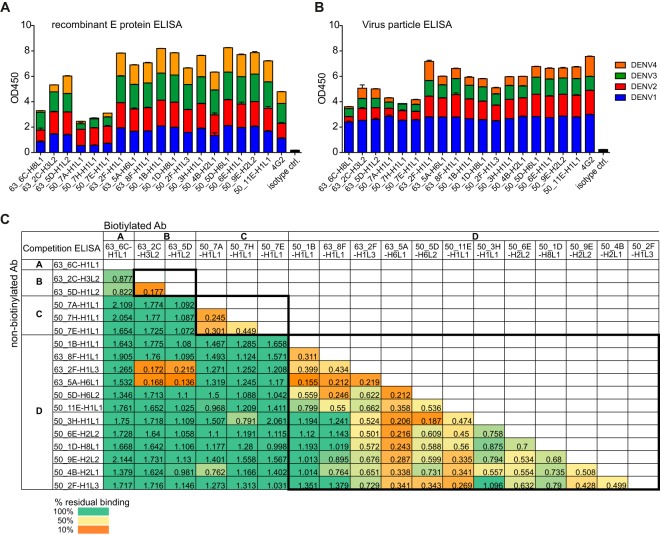
Binding groups of human plasmablast-derived antibodies. (A and B) ELISA of dengue virus-specific antibodies to rE protein (A) and to whole viral particles (B) of all four DENV serotypes. (C) One-way competition ELISA. Nonlabeled antibodies were added in 100-fold excess over biotinylated antibodies to compete for binding sites on the captured rE protein. The values are normalized to antibody binding in the absence of competition. Blocking was defined as reduction in absorbance readings by at least 70%.

Previously, we described the generation of a panel of human monoclonal antibodies from the plasmablasts of two naturally infected dengue patients by single B cell PCR cloning. The majority of these plasmablast-derived antibodies were dengue virus specific, and the primary target was found to be the E protein (7). Here, we further characterized a subset of clonally distinct antibodies that were selected based on good binding to both E protein and virus particles (Fig. 1A and B). The relative ability of each antibody to block binding of all other antibodies individually in a competition ELISA was tested to identify common antibody epitopes (Fig. 1C). Unlabeled antibodies were used in 100-fold excess over biotinylated antibodies, and successful competition was defined as a reduction of the normalized streptavidin-HRP signal by at least 70%. Four main groups of antibodies, designated A, B, C, and D, were identified based on this competition assay. The largest group, D, comprised more than 50% of the antibodies tested (Fig. 1C). Interestingly, biotinylated antibodies from groups A, B, and C appeared to promote binding of antibodies in the same or other groups, as illustrated by a value higher than 1 (Fig. 1C, dark green cells), which is the maximal binding of each biotinylated antibody in the absence of competition.

### The acute-phase antibody repertoire is dominated by fusion loop-specific antibodies.

**FIG 2:**
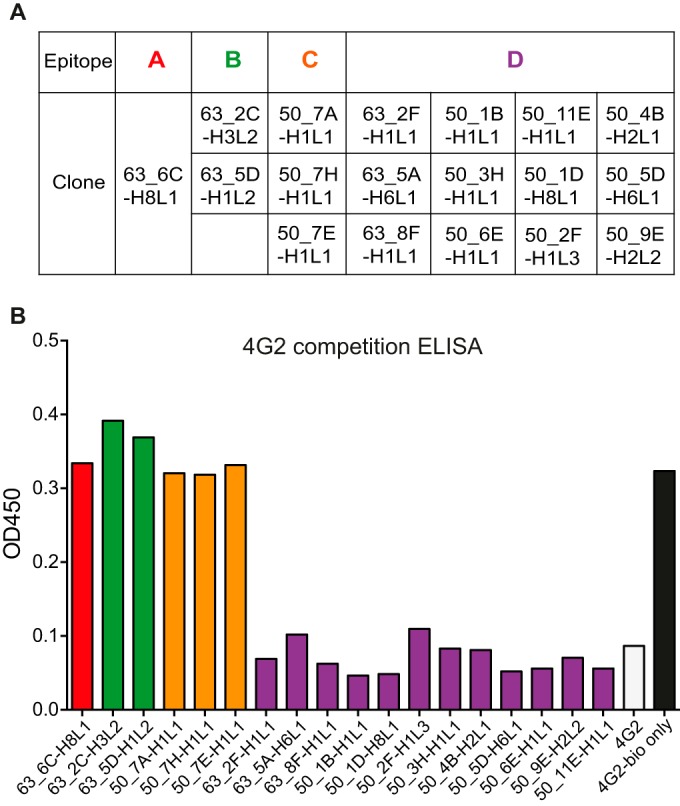
Group D antibodies are DENV fusion loop specific. (A) Summary of antibody clones in each of the four binding groups A, B, C, and D and the color code for each group. (B) 4G2 competition ELISA. Nonlabeled antibodies were added in 100-fold excess over biotinylated 4G2 to compete for binding sites on the surface of the captured rE.

In order to identify the footprint of these antibodies on the E protein, another competition ELISA was performed. The same panel of unlabeled antibodies, ordered according to their respective groups in Fig. 2A, was used to compete with the well-characterized mouse monoclonal antibody 4G2 (21). The described epitope of 4G2 comprises amino acids G104, G106, and L107, which lie in the fusion loop of the E protein (22, 23). Competition for binding sites on the immobilized recombinant E protein revealed that 4G2 competed with 12 out of 18 antibodies (Fig. 2B). These antibodies comprised the dominant group D, and antibodies in this group thus share an epitope with 4G2 in the fusion loop. The dominance of fusion loop-specific antibodies is consistent with the previous literature (24, 25). In addition, 2 out of 18 antibodies (11%) and 3 out of 18 antibodies (17%) clustered in groups B and C, respectively. Even though the total number of antibodies analyzed in this study is too small to draw general conclusions, the finding indicates that epitopes of groups B and C could also be immunodominant during natural infection.

### Epitope-mapping studies reveal that non-solvent-exposed residues of the E protein are critical antibody binding sites.

**FIG 3:**
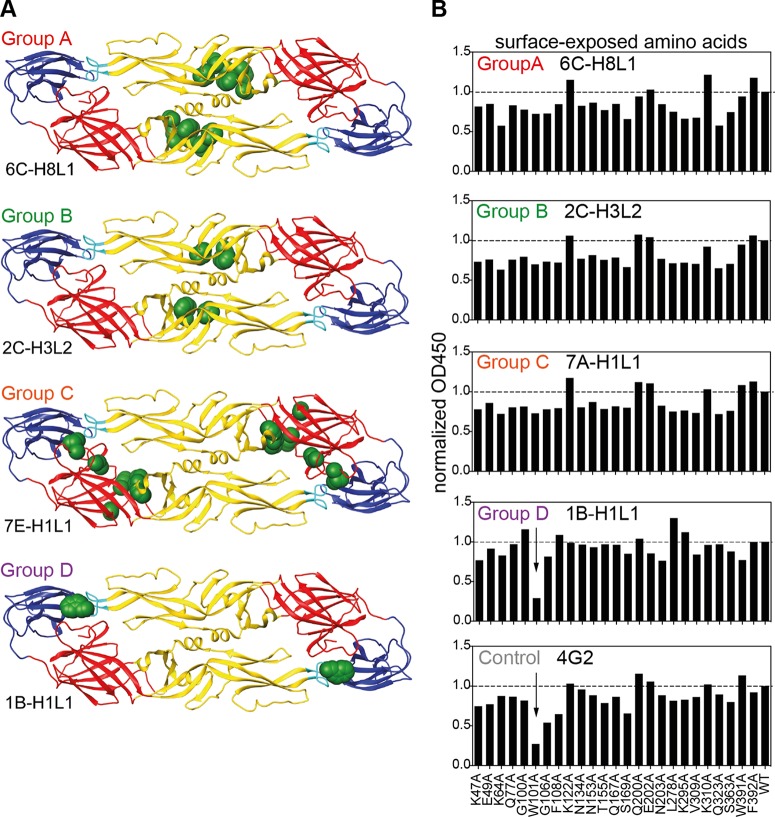
Groups of antibodies bind to distinct epitopes in EDI or EDII. (A) Amino acid contact residues engaged by antibodies were identified by shotgun mutagenesis mapping. DENV prM/E mutants were expressed in HEK-293T cells, and binding by test antibodies was detected by a fluorescent secondary antibody, normalizing the results to the mean fluorescence intensity. (B) Sandwich ELISA with rE proteins containing alanine replacement mutations in surface-exposed amino acids. The values were normalized to unmutated rE.

To further dissect the epitopes of antibodies in groups A, B, C, and D, an alanine scanning mutagenesis approach was used (Fig. 3A and B) (26). For these studies, comprehensive mutation libraries of DENV-3 or DENV-4 prM/E proteins were expressed in HEK-293T cells, and binding of the antibodies was detected by flow cytometry. We selected a representative antibody from each of the four groups for these epitope-mapping studies. Residues critical for each antibody epitope were initially identified as those where prM/E mutations resulted in low reactivity for the antibody of interest (20 to 30% relative to wild-type DENV prM/E) yet greater than 60% of wild-type binding by a control MAb. Antibodies representative of groups A and B both bound to EDII. For group A antibody 6C-H8L1, we identified residues D215 and P217 as critical for binding; for group B antibody 6C-H8L1, we also identified D215 and P217, as well as residues H209, W212, and A267. The group C antibody 7E-H1L1 was mapped to EDI with critical residues C30, V151, R186, G279, H280, and K282 (Fig. 3A; see Fig. S2A in the supplemental material). Intriguingly, all the contact residues for group A, B, and C antibodies identified in EDI and EDII are poorly exposed in the available E protein dimer crystal structures (Fig. 3A).

To help confirm the locations of these epitope residues, we converted group B antibody 2C-H3L2 to a Fab, which was then screened on the Ala scan mutation library. Conversion of antibody to a Fab weakens binding relative to the full antibody, which we have found allows the identification of additional epitope residues compared to the full-length MAb. Using the Fab, we identified two additional residues as critical for 2C-H3L2 binding in proximity to the original residues identified (see Fig. S2B in the supplemental material). No residues were identified on the surface of the E protein, and similar to the residues identified for the full antibody, the residues identified for the 2C-H3L2 Fab were not exposed on the viral surface in the available E dimer crystal structures. This supports the apparently poorly exposed residues identified here as contributing to the antibody epitopes.

Additionally, for the mutations at poorly exposed residues, we have compared their binding by several antibodies with epitopes at different sites on the E protein surface (see Table S2 in the supplemental material). The binding of these antibodies was not decreased by residues identified as epitopes for the group A, B, or C antibodies. We note in particular that binding of the quaternary antibody 5J7 (27) lies on the E protein surface at the DI-DII interface close to the mutations identified on the underside of the E protein for group C antibody 7E-H1L1. However, binding by 5J7 was unaffected by any of these mutations on the underside, further suggesting that the epitope residues identified here for group A, B, C, and D antibodies do not perturb the global structure of the E protein.

Mapping of the group D antibodies identified residues associated with MAbs that bind to the DENV fusion loop, W101, G106, L107, and F108 (Fig. 3; see Fig. S3 in the supplemental material). In addition, MAb 1D-H4L1 included residue G78 in the bc loop, adjacent to the fusion loop on DII, a site that includes highly neutralizing epitopes (see Fig. S2 in the supplemental material) (28).

E proteins expressed recombinantly in HEK-293 cells might not form dimers efficiently, or the structure might be different than on virions. This is important, since it has been suggested recently that E dimer-specific antibodies are abundant in the plasmablast response (2). To address this potential limitation of the HEK cell-based screen, we employed an additional ELISA-based epitope-mapping approach using a panel of recombinant E proteins with alanine replacement mutations in surface-exposed amino acids that are commonly recognized by human antibodies (2, 25) (Fig. 3B). A sandwich approach was used to increase the concentration of E protein dimers, and the assay was validated with previously published E dimer-specific antibodies (29) (see Fig. S1 in the supplemental material). This assay confirmed that group D antibodies bound to the fusion loop. The assay also suggested that none of the group A, B, and C antibodies bound to virus surface-exposed epitopes that were described previously for plasmablast-derived antibodies (2). Of note, only correctly folded E protein mutants that were recognized by the mixture of positive-control antibodies were included in this analysis. Potential antibody binding sites that could not be mutated due to misfolding of the E protein in drosophila cells (L107A, E161A, I162A, and S274A) were therefore not addressed.

### Antibody binding is influenced by pH.

**FIG 4:**
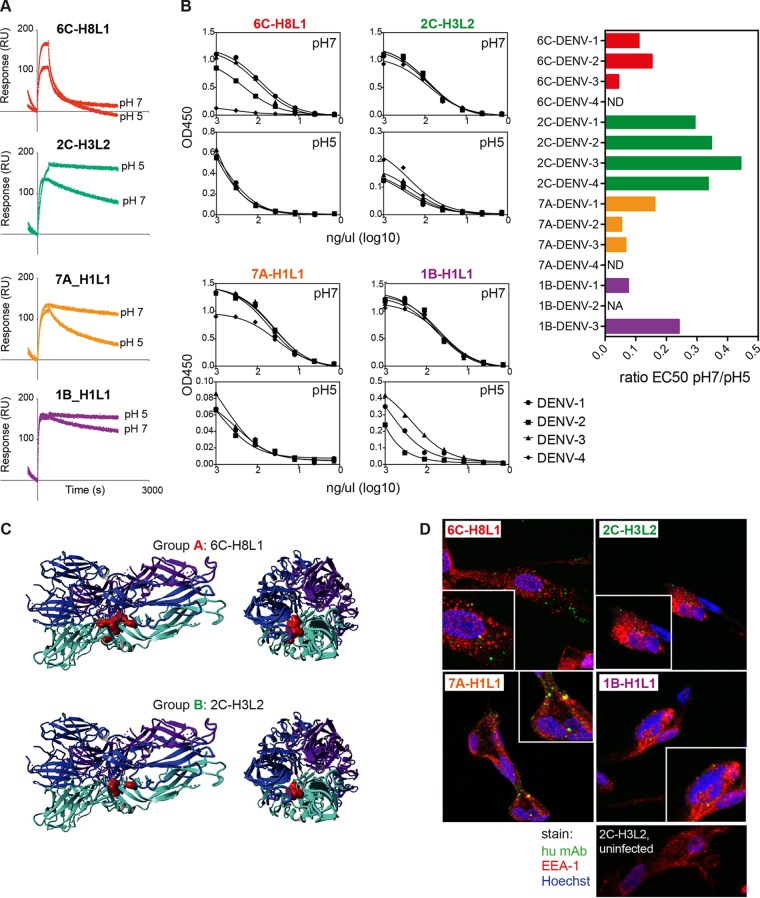
Antibody groups show different stabilities at low pH. (A) The stability of antibody binding at pH 5 or pH 7 was assessed by surface plasmon resonance. A representative sensorgram from an antibody from each linkage group is shown. (B) Stability of antibody binding at pH 5 or pH 7 for the same antibodies was tested in a sandwich ELISA (see Materials and Methods) for all four serotypes. The ratios of EC_50_ values for pH 7 to the EC_50_ values for pH 5 are shown for each tested antibody. ND, not done; NA, not applicable, since the curve fit for pH 5 was ambiguous. (C) Epitopes of antibodies 6C-H8L1 and 2C-H3L2 illustrated on the trimeric form of the E protein. The three E proteins are shown in blue, light blue, and purple, and the epitope is indicated in only one of the E proteins. (D) BHK-21 cells 7 min after uptake of the indicated antibodies complexed with DENV-1. Anti-human IgG (green) and anti-EEA-1 (red) were used to detect complexes in the early endosomes (yellow). The insets are magnified areas of the main images.

To deduce the possible functions of these antibodies, we tested their sensitivity to pH changes by surface plasmon resonance (SPR). The antibodies were immobilized on SPR sensor chips, followed by the flowing of E protein at either pH 7 or pH 5. The dissociation kinetics of the antibodies at either pH 7 or pH 5 were compared. While antibodies from groups B and D demonstrated increased stability at pH 5, the reverse was true for antibodies from group C. These antibodies showed decreased stability at pH 5. Group A antibodies were not sensitive to pH changes and displayed relatively rapid dissociation at both pHs (Fig. 4A). To confirm the pH-dependent binding, we also employed the sandwich ELISA described for Fig. 3B and immobilized E proteins that were first equilibrated at pH 5 or 7, followed by the addition of antibodies at the respective pH (Fig. 4B). Binding at pH 5 was generally lower, possibly due to the less efficient binding of E protein to the plates at this pH. Nevertheless, a less steep loss of binding with decreasing concentration showed that antibody 2C-H3L2 (group B) was the most stable at pH 5 compared to pH 7 (Fig. 4B), and this was true for all four serotypes. However, similar to epitope exposure on E dimers (Fig. 3A), the epitopes of groups A, B, and C were also not surface exposed in the endosome-associated postfusion trimeric E protein structure (Fig. 4C). Interestingly, the stability of antibody 1B-H1L1 (group D) at pH 5 was serotype dependent, despite the completely conserved epitope of the antibody (Fig. 4B). To test whether antibodies could be detected in the early endosome after uptake of virus-antibody complexes, we incubated BHK-21 cells with DENV-1–antibody complexes for 7 min and detected the complexes with fluorescent anti-human IgG antibodies (Fig. 4D). While not all complexes colocalized in the EEA-1-expressing early endosome, there was evidence for colocalization for at least three of the four antibodies. However, there was no obvious correlation with pH stability.

### Fusion loop-specific antibodies are broadly cross-neutralizing but poor in protection, whereas group B antibodies are poor neutralizers but superior in protection.

**FIG 5:**
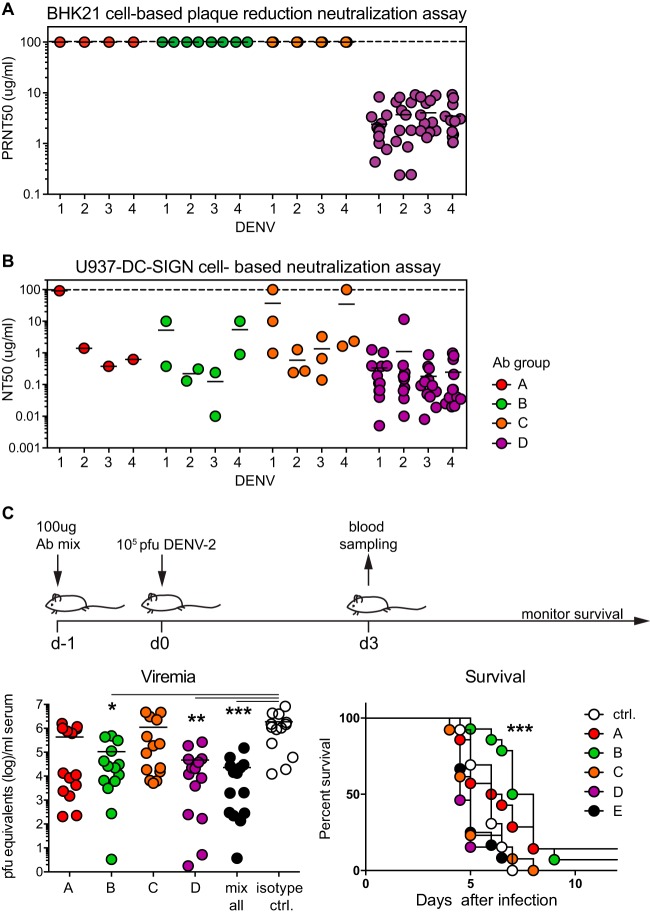
Group B antibodies promote survival despite low *in vitro* neutralizing capacity. (A) BHK21 cell-based PRNT of all Abs against all four DENV serotypes ordered according to color-coded groups A to D. The highest concentration tested was 100 μg/ml, indicated by the dashed line. Antibodies that were not neutralizing at 100 μg/ml were arbitrarily assigned a PRNT_50_ value of 100 μg/ml. Each dot represents one antibody. (B) U937-DC-SIGN cell-based neutralization assay of all the Abs against all four DENV serotypes, ordered according to color-coded groups A to D. Each dot represents one antibody (7). (C) *In vivo* protection assay in AG129 mice as illustrated in the treatment scheme. Each mouse was treated i.v. with a total of 100 μg of a mixture of Abs from each group (1 for group A, 2 for group B, 3 for group C, 12 for group D, and 18 for group E [A, B, C, and D]). Viremia was measured 3 days after infection with DENV-2, and mouse survival was monitored until day 12. A Kruskal-Wallis test was used to compare the groups, and a *P* value of <0.05 was considered statistically significant. Survival statistics were calculated using a log-rank Mantel-Cox test and applying a Bonferroni-corrected *P* value of 0.01 as the threshold for statistical significance, considering five comparisons of isotype control versus treatment.

The envelope protein is the major target of neutralizing antibodies following infection. Neutralization was performed to draw a link between binding of an antibody to a particular epitope and the capacity to block infection. Two assays were employed to account for potential differences in antibody-neutralizing capacity depending on the host cell and the expression of the virus receptor DC-SIGN (30). The historical gold standard for virus neutralization is a PRNT with BHK21 cells. An alternative is a flow cytometry-based assay using U937 cells expressing DC-SIGN to facilitate infection (7). The fusion loop antibodies of group D displayed moderate cross-neutralization capacities across all four dengue virus serotypes in both assays (Fig. 5A and B). While antibodies from groups A, B, and C were not neutralizing by PRNT (Fig. 5A), they showed variable and weak neutralization in the flow cytometry-based assay (Fig. 5B) (7).

To understand the protective capacity of group A to D antibodies and their potential relevance in resolving an infection *in vivo*, we employed a lethal DENV-2 mouse infection model (31). Interestingly, antibodies from groups B and D reduced viremia 10- and 100-fold, respectively, but only group B antibodies increased survival of the animals significantly. In fact, group D antibodies, which led to a higher reduction in viremia than group B antibodies, tended to negatively affect survival. Viremia in animals treated with group C was comparable to that in the isotype control-treated group, and group C antibodies also did not impact survival. Finally, animals treated with a combination of antibodies from all four groups (group E) had a 100-fold reduction in the viremia load, but the reduction did not promote survival (Fig. 4C). These experiments demonstrated that *in vitro* neutralizing capacity does not necessarily correlate with the capacity to reduce viremia and that lower viremia does not necessarily correlate with longer survival.

## DISCUSSION

The ability to elicit neutralizing antibodies to prevent reinfection is a key aspect of immune memory. The human memory response to natural dengue virus infection contains cross-reactive, mostly weakly neutralizing antibodies; serotype-specific potent neutralizing antibodies; prM-specific antibodies with the potential to both neutralize and enhance infection; and antibodies against the nonstructural protein NS1 (1, 3, 4, 32). To date, the literature has largely focused on deciphering the epitopes of potently neutralizing antibodies. However, large quantities of less potently neutralizing antibodies produced by plasmablasts at a time of infection when viremia is already declining could have an impact on disease progression, and there is a need to characterize these antibodies in greater detail. Dejnirattisai et al. recently described potently neutralizing, serotype-cross-reacting antibodies isolated from plasmablasts (2). These antibodies were E protein dimer specific (see Fig. S1 in the supplemental material). Not all patients, however, seem to produce such antibodies at high frequency, and the antibodies were isolated from only three out of seven patients in the Dejnirattisai et al. study. This could explain why such antibodies were not among the panel analyzed here. The panel was selected based on good binding in both E protein and virus particle ELISA, which represents the majority of antibodies in our case (88 to 100% of DENV-specific antibodies) (7).

In the context of dengue virus infection, neutralizing-antibody titers do not seem to suffice as a correlate of protection, and we therefore also tested plasmablast-derived antibodies *in vivo*. We had tested 9 of the 18 antibodies previously in a mouse model of infection and found that the protective capacity was increased for the serotype of the previous infection of the plasmablast donor compared to the serotype of the ongoing infection, pointing to the memory B cell origin of the plasmablasts (7). The readout of that study was viremia, which is often a good indicator of the *in vivo* protective capacity of an antibody. In patients, however, viremia is not always associated with disease severity (33, 34). Secondary infection is associated with a higher risk of severe disease. However, there is no clear difference in viremia between primary and secondary infections, at least not in all serotypes (35). We have found previously in mouse models that viremia in the blood does not correlate with viremia in all organs (36). A higher viral load in the lymphatic organs might in fact help to induce an efficient immune response and faster clearance of the virus, as shown for other viruses (37, 38). In the current study, we therefore aimed to address the protective capacity of antibodies, using not only viremia but also survival as a readout.

Antibodies from groups A and B reduced viremia between 10- and 100-fold, whereas antibodies from group C did not, despite equally poor neutralization capacities of groups A, B, and C *in vitro*. It is important to note that although viremia was reduced in mice treated with group D antibodies, reduction in viremia did not correlate with longer survival. The epitopes of the non-fusion loop antibodies were mapped to nonexposed residues. We observed that binding by antibodies from groups A, B, and C “promoted” binding of other antibodies. Dengue virus is known to be flexible and to assume variable structures at different temperatures, in contrast to other flaviviruses, such as Zika virus (39). This flexibility promotes exposure of epitopes (40) that could then potentially be “locked” by group A, B, and C antibodies, allowing access to other antibodies. Hence, epitopes that are apparently hidden in structures that were solved under one specific condition may still be accessible to antibodies at increased temperature or different pH. This accessibility might be serotype dependent, as suggested by differential binding of the fusion loop-specific antibody 1B-H1L1 to DENV-1, -2, and -3 at pH 5 (Fig. 4B).

Overall, these observations suggested that neutralization mechanisms other than direct blocking of virus attachment to host cells are crucial for the protective capacity of antibodies. While mouse models cannot replicate all aspects of a human infection, *in vivo* studies are useful to reveal aspects that are potentially relevant for protection in patients and that cannot be observed in *in vitro* assays. Interestingly, survival seemed to correlate with the pH stability of the antibodies (group C Abs were less stable at pH 5 and not protective compared to group B Abs, which were more stable at pH 5 and more protective). The neutralizing effect could potentially involve virus fusion inhibition in the endosome, as proposed for West Nile virus-specific antibody E16 (41). However, while blocking of the fusion event may be relevant, fusion loop specificity does not seem to be sufficient to effectively block viremia and prolong survival *in vivo*, as is evident from the data for group D antibodies. More studies are needed to address the relevance of antibody stability and binding at low pH and the possible blocking of E protein dimer-to-trimer transformation in the context of the systemic virus load and survival.

None of the antibody groups fully protected mice at the concentration of 100 μg that was used per mouse, and these antibodies, when tested in individual groups, cannot be considered very potent *in vivo*. Nevertheless, the differential ability of the antibodies to decrease viremia and to prolong survival provides new insight into possible mechanisms of antibody-mediated protection, and this could help to define correlates of protection.

## Supplementary Material

Supplemental material
