# Supplementary material for: Protective Capacity of the Human Anamnestic Antibody Response during Acute Dengue Virus Infection
Source: J Virol. 2016 Nov 28;90(24):11122–31. doi: 10.1128/JVI.01096-16 (PMC5126370; doi:10.1128/JVI.01096-16)
Supplement: Supplemental material [file supp_90_24_11122__index.html]

Supplemental material 

# Protective Capacity of the Human Anamnestic Antibody Response during Acute Dengue Virus Infection

## Supplemental material

**Files in this Data Supplement:**

- Supplemental file 1 -

  Fig. S1 (Validation of the anti-V5-tag sandwich ELISA for detection of E dimer-specific antibodies.) Fig. S2 (Control experiments for epitope mapping using mutant prM/E protein libraries expressed in HEK cells.) Fig. S3 (Epitope mapping of six group D antibodies.) Table S1 (GenBank accession numbers for heavy and light chains of all antibodies described in this study.) Table S2 (Summary antibody binding data.)

  PDF, 1.2M
