# Supplementary material for: Protective Capacity of the Human Anamnestic Antibody Response during Acute Dengue Virus Infection
Source: J Virol. 2016 Nov 28;90(24):11122–31. doi: 10.1128/JVI.01096-16 (PMC5126370; doi:10.1128/JVI.01096-16)
Supplement: Supplemental material [file JVI.01096-16_zjv999182190s1.pdf]

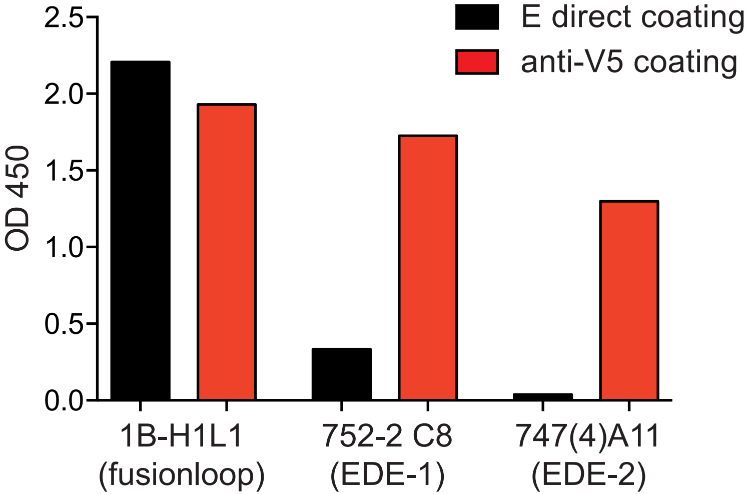

SFig.1: Validation of the anti-V5-tag sandwich ELISA for detection of E dimer-specific antibodies. Recombinant DENV-2 E protein was coated directly on ELISA plates (black bars) or plates were coated with anti-V5-tag antibody, followed by the addition of recombinant E protein that contains a V5 tag (red bars). Antibodies tested and their respective epitopes in parenthesis are indicated on the x axis. EDE: E dimer epitope. Bars are means of two replicates.

A

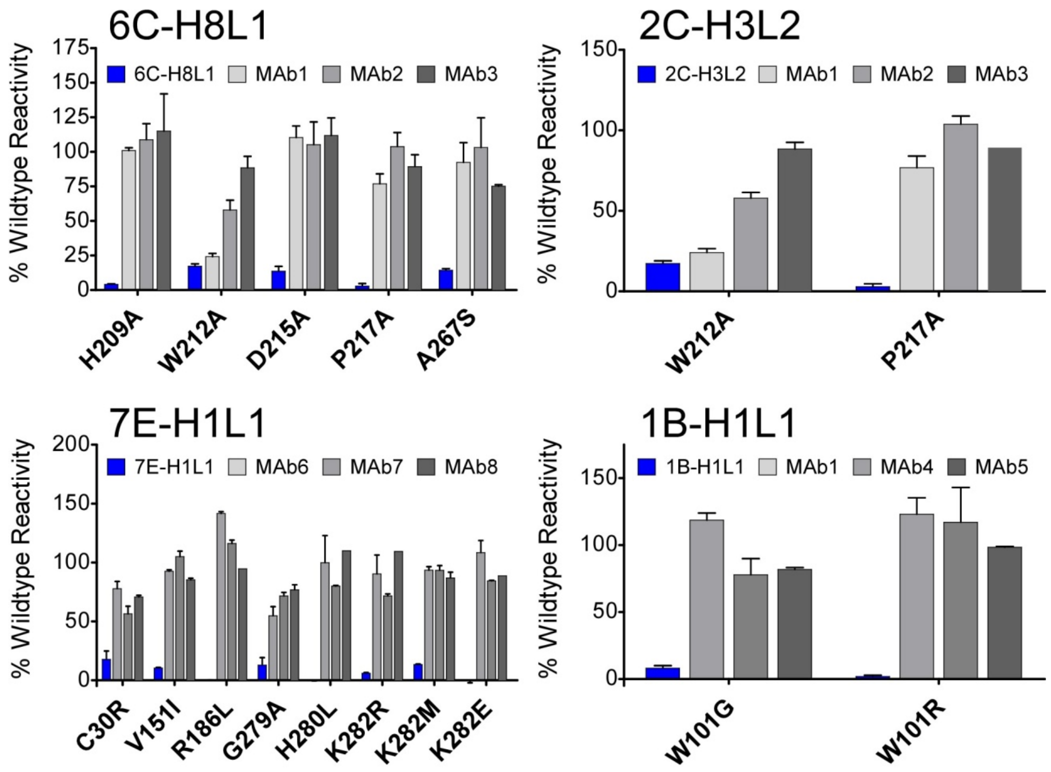

B

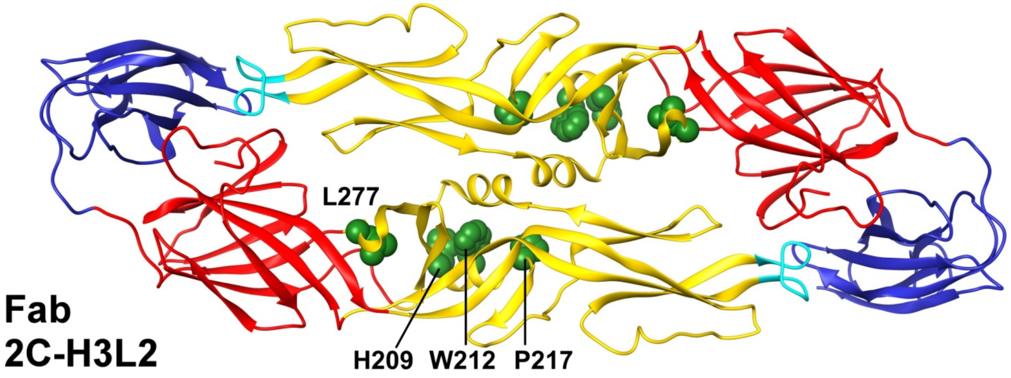

SFig. 2: Control experiments for epitope mapping using mutant prM/E protein libraries expressed in HEK cells. A) Binding results for candidate mAbs (blue bars) in comparison to control antibodies (grey bars). B) MAb 2C-H3L2 from Group B was converted to a Fab by papain digestion, and screened on the DENV4 Ala-scan mutation library. This identified W212 and two additional residues as critical for binding (H209 and L277) that were not identified for the MAb. None of the residues are predicted to be exposed on the surface of the E protein in the crystal structure PDB id 1OAN.

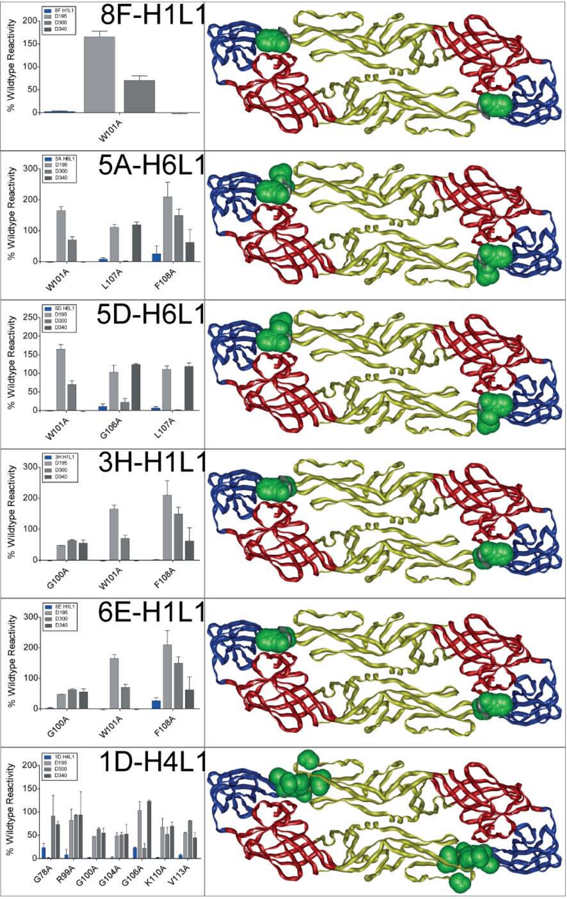

SFig.3: Epitope mapping of six group D antibodies that was done in addition to 1B-H1L1, which is shown as a representative antibody in Fig.4.

**Antibody ID**	**Antibody chain**	**GenBank Accession number**
Human10_63-2C-H3	Heavy chain	KX858673
Human10_63-2C-L2	Light chain	KX858674
Human10_63-2F-H1	Heavy chain	KX858675
Human10_63-2F-L1	Light chain	KX858676
Human10_63-5A-H6	Heavy chain	KX858677
Human10_63-5A-L1	Light chain	KX858678
Human10_63-5D-H1	Heavy chain	KX858679
Human10_63-5D-L2	Light chain	KX858680
Human10_63-6C-H8	Heavy chain	KX858681
Human10_63-6C-H1	Light chain	KX858682
Human10_63-8F-H1	Heavy chain	KX858683
Human10_63-8F-L1	Light chain	KX858684
Human10_50-1B-H1	Heavy chain	KX858685
Human10_50-1B-L1	Light chain	KX858686
Human10_50-1D-H8	Heavy chain	KX858687
Human10_50-1D-L1	Light chain	KX858688
Human10_50-2F1	Heavy chain	KX858689
Human10_50-2F3	Light chain	KX858690
Human10_50-3H-H1	Heavy chain	KX858691
Human10_50-3H-L1	Light chain	KX858692
Human10_50-4B-H2	Heavy chain	KX858693
Human10_50-4B-L1	Light chain	KX858694
Human10_50-5D-H6	Heavy chain	KX858695
Human10_50-5D-L2	Light chain	KX858696
Human10_50-6E-H1	Heavy chain	KX858697
Human10_50-6E-L1	Light chain	KX858698
Human10_50-7A-H1	Heavy chain	KX858699
Human10_50-7A-L1	Light chain	KX858700
Human10_50-7E-H1	Heavy chain	KX858701
Human10_50-7E-L1	Light chain	KX858702
Human10_50-7H-H1	Heavy chain	KX858703
Human10_50-7H-L1	Light chain	KX858704
Human10_50-9E-H2	Heavy chain	KX858705
Human10_50-9E-L2	Light chain	KX858706
Human10_50-11E-H1	Heavy chain	KX858707
Human10_50-11E-L1	Light chain	KX858708

Supplementary Table 1: GenBank accession numbers for heavy and light chains of all antibodies described in this study

DENV3					
	**7E-H1L1**	**5J7**	**D11C**	**Budding**	**Infectivity**
C30R	18 (7)	50 (6)	63 (9)	1	0
V151I	10 (1)	88 (12)	122 (6)	126	96
R186L	0 (0)	92 (3)	126 (22)	262	1
G279A	13 (6)	73 (4)	68 (5)	34	46
H280L	0 (0)	97 (2)	104 (1)	15	0
K282R	6 (0)	110 (2)	101 (3)	131	26
K282M	13 (1)	125 (3)	100 (0)	62	0
K282E	0 (2)	80 (15)	103 (5)	3	0

DENV4							
	**2C-H3L2**	**6C-H8L1**	**5A-H6L1**	**1C19**	**3H4**	**Budding**	**Infectivity**
W101A	83 (0)	111 (9)	-2 (0)	165 (12)	0 (1)	4	1
L107A	116 (6)	145 (1)	8 (2)	111 (9)	119 (4)	-1	0
F108A	119 (8)	141 (5)	25 (13)	210 (47)	62 (21)	48	0
H209A	31 (1)	4 (0)	93 (6)	109 (6)	115 (14)	204	19
W212A	17 (2)	17 (2)	44 (5)	58 (4)	88 (4)	18	0
D215A	34 (3)	134 (4)	109 (7)	105 (8)	112 (6)	100	0
P217A	3 (2)	3 (2)	81 (6)	103 (5)	89 (4)	66	4
A267S	55 (13)	14 (1)	82 (3)	103 (11)	75 (1)	97	56

Supplemental Table 2. Summary antibody binding data. Summary binding data for anti-DENV plasmablast and control antibodies is shown, mapped on the DENV3 or DENV4 prM/E mutation libraries. The control antibodies bind to distinct regions of the E protein, at the fusion loop (3H4, Smith et al, 2013, mBio; D11C, Costin et al, 2013), the bc loop (1C19, Smith et al, 2013, mBio), and a quaternary epitope whose critical residues lie at the EDI/EDII interface (5J7, Messer et al 2016 J. Virol). Antibody reactivities for each mutant are expressed as percent of binding to the parental wild-type prM/E, with ranges (half of the maximum minus minimum values) in parentheses. At least two replicate values were obtained for each experiment. Also shown for each prM/E variant are values for virus budding and infectivity, obtained as described previously (Christian et al., 2013). All values are expressed as a percentage of antibody binding to wild-type DENV prM/E, or of wild-type values for budding and infectivity.
